# Cardiovascular Risk Score, Cognitive Decline, and Dementia in Older Mexican Americans: The Role of Sex and Education

**DOI:** 10.1161/JAHA.113.004978

**Published:** 2013-04-24

**Authors:** Adina Zeki Al Hazzouri, Mary N. Haan, John M. Neuhaus, Mark Pletcher, Carmen A. Peralta, Lenny López, Eliseo J. Pérez Stable

**Affiliations:** 1 Department of Epidemiology & Biostatistics, School of Medicine, University of California San Francisco, San Francisco, CA (A.Z.A.H., M.N.H., J.M.N., M.P.); 2 Division of General Internal Medicine, School of Medicine, University of California San Francisco, San Francisco, CA (C.A.P., E.J.P.S.); 3 Harvard Medical School, Boston, MA (L.L.)

**Keywords:** aging, cardiovascular disease risk factors, epidemiology, risk score

## Abstract

**Background:**

The purpose of this study was to examine the associations of cardiovascular disease (CVD) risk with cognitive decline and incidence of dementia and cognitive impairment but not dementia (CIND) and the role of education as a modifier of these effects.

**Methods and Results:**

One thousand one hundred sixteen Mexican American elderly were followed annually in the Sacramento Area Latino Study on Aging. Our sex‐specific 10‐year CVD risk score included baseline age, systolic blood pressure, total cholesterol, high‐density lipoprotein, smoking, body mass index, and diabetes. From adjusted linear mixed models, errors on the Modified Mini–Mental State Exam (3MSE) were annually 0.41% lower for women at the 25th percentile of CVD risk, 0.11% higher at the 50th percentile, and 0.83% higher at the 75th percentile (*P* value of CVDrisk×time <0.01). In men, 3MSE errors were annually 1.76% lower at the 25th percentile of CVD risk, 0.96% lower at the 50th percentile, and 0.12% higher at the 75th percentile (*P* value of CVDrisk×time <0.01). From adjusted linear mixed models, the annual decrease in the Spanish and English Verbal Learning Test score was 0.09 points for women at the 25th percentile of CVD risk, 0.10 points at the 50th percentile, and 0.12 points at the 75th percentile (*P* value of CVDrisk×time=0.02). From adjusted Cox models in women, compared with having <6 years of education, having 12+ years of education was associated with a 76% lower hazard of dementia/CIND (95% CI, 0.08 to 0.71) at the 25th percentile of CVD risk and with a 45% lower hazard (95% CI, 0.28 to 1.07) at the 75th percentile (*P* value of CVDrisk×education=0.05).

**Conclusions:**

CVD risk score may provide a useful tool for identifying individuals at risk for cognitive decline and dementia.

## Introduction

Cardiovascular disease (CVD) and its risk factors remain the most common causes of morbidity and mortality in the United States.^1–2^ Specific risk factors such as type 2 diabetes,^3^ hypertension,^4^ obesity,^5–6^ and high lipids^7^ have been associated with greater cognitive decline and risk of developing dementia. These risk factors are modifiable and constitute potential targets for interventions to prevent or delay cognitive impairment.

There is increasing evidence that summary scores measuring CVD risk are predictive of cognitive decline.^8,10^ Some prior research has focused on the Framingham Stroke Risk Profile (FSRP).^10–13^ The FSRP does not cover the whole range of CVD, and the majority of work has examined cross‐sectional rather than longitudinal associations. Furthermore, no prior work was conducted in low‐income minority populations such as Mexican Americans. Compared with non‐Latino whites, Mexican Americans are more burdened with CVD risk factors such as obesity^14^ and type 2 diabetes^15^ and show lower awareness, treatment, and control of certain CVD risk factors.^16^ As such, the collective effect of multiple cardiovascular risk factors on cognitive outcomes among this disadvantaged group may be different from other aging racial/ethnic groups.

Cardiovascular exposures are patterned by markers of socioeconomic status (SES) such that lower SES is often associated with worse cardiovascular profile.^17^ Education is a measure of SES and plays a unique role in shaping cognitive function.^18–20^ Recent results from our research group have shown that higher educational attainment is associated with slower rates of cognitive decline and lower incidence of dementia in US Hispanics.^21–23^ Other measures of SES may be less predictive in older populations such as household income, which often declines with retirement. Socioeconomic status has been shown to modify the effect of exposures on health outcomes^24–25^ including that of cardiovascular exposures on cognitive outcomes of a middle‐aged cohort.^26^ However, the effect of education on the associations between cardiovascular exposures and cognitive health at old age remains largely unexplored, particularly in minority populations.

In the present study, we sought to determine the associations of CVD risk with cognitive decline and incidence of dementia and cognitive impairment without dementia (CIND) in a cohort of elderly Mexican Americans followed over 10 years. Another objective of this study was to examine the role of education in the associations of CVD risk with cognitive decline and dementia/CIND.

## Methods

### Study Population

Participants in this study were from the Sacramento Area Latino Study on Aging (SALSA). SALSA is a prospective cohort study of 1789 community‐dwelling older Mexican Americans aged 60 to 101 years at baseline in 1998–1999. Every 12 to 15 months, biological and clinical data were collected on participants in home visits for a maximum of 6 follow‐ups. The SALSA study has been approved by the institutional review boards at the University of Michigan, the University of California, San Francisco, and the University of California, Davis. Details on the study design have been published elsewhere.^27^ Of the 1789 participants, we excluded those with baseline CVD (n=496) or with missing data on any of the component variables of the CVD risk score, our predictor of interest (n=127). Of the remaining, we further excluded those with a baseline diagnosis of dementia/CIND (n=50). A total of 1116 thus constituted the final sample for the present analysis.

### Measures

#### Cardiovascular risk factors and other covariates

At baseline, systolic blood pressure (SBP) and diastolic blood pressure (DBP) were measured using a digital blood pressure monitor. Fasting blood was collected and analyzed for lipids including high‐density lipoprotein (HDL; mg/dL) and total cholesterol (mg/dL). Standing height and weight were measured, and body mass index (BMI; kg/m^2^) was calculated as weight/(height×height). Prevalent type 2 diabetes was ascertained as self‐report of a physician diagnosis, use of diabetes medication, or a fasting glucose level ≥126 mg/dL. Hypertension was ascertained as report of a physician diagnosis, use of hypertension medication, a systolic blood pressure >140 mm Hg, or a diastolic blood pressure >90 mm Hg. We classified hypertension into 3 categories as “no hypertension,” “hypertensive but not taking antihypertensive medications,” and “hypertensive and taking antihypertensive medications.” Participants reported their smoking status (current, former, or never) and their country of birth (US‐ or Mexican‐born).

#### Socioeconomic status

Participants in SALSA reported various measures of socioeconomic status including education, major lifetime occupation, and old‐age household income. Because of the particularly important role of education in relation to cognitive health, we focused on education in the present analysis. Participants reported the years of education they had completed, which was categorized as <6 (low), ≥6 and <12 (middle), and ≥12 (high) years.

#### CVD risk score estimation

CVD risk, our predictor of interest, measures the 10‐year probability of developing CVD during the SALSA study period. Incident CVD included myocardial infarction (MI), angina, stroke, heart failure, atrial fibrillation, coronary catheterization, and death from any of these CVD events. Deaths were identified using online obituaries; review of the Social Security Death Index, vital statistics data files, and death certificates; and interviews with family members. We used codes from the Tenth Revision of the International Statistical Classification of Diseases to classify CVD as a cause of death if listed anywhere on the death certificate.

Following criteria published by Sullivan and colleagues,^28^ we estimated the 10‐year predicted CVD risk of each individual using sex‐specific Cox proportional hazard models. These Cox models incorporated baseline age, SBP, total cholesterol, HDL, smoking status, BMI, and type 2 diabetes and had survival time to incident CVD as an outcome over a 10‐year period. We multiplied our CVD risk by a factor of 100 (range, 0% to 100%), and that was interpretable as percent predicted CVD risk and was modeled as continuous. Variables included in our CVD risk estimation were identified by the Framingham Heart Study as components of the general CVD risk score.^29^ Given their clinical importance, these risk factors are applicable to nonwhite populations.^30^

#### Cognitive function and dementia/CIND

For all participants, cognitive function was assessed using the Modified Mini–Mental State Exam (3MSE) and the Spanish English Verbal Learning Test (SEVLT). The 3MSE is a 100‐point test of global cognitive function and was validated and field‐tested in both English and Spanish. Compared with the Mini–Mental State Exam (MMSE), the 3MSE shows better reliability, test–retest properties, sensitivity, and specificity and fewer ceiling effects.^31–32^ The SEVLT is a 15‐point verbal memory recall test with four 15‐word memory trials, an interference list, followed by a fifth trial that is usually used as the test score.^33–34^ SEVLT was developed for use in SALSA^34^ and has been validated in both English and Spanish and has been used in other studies. Higher scores on both tests indicate better cognitive function. Both cognitive tests were administered at all study visits.

A multistage screening process was used for the diagnosis of incident dementia or CIND cases over the 10‐year follow‐up period. In the first stage, the 3MSE and SEVLT were administered. If participants scored below the 20th percentile on either test or if their scores declined by >8 or >3 points, respectively, from the previous examination, participants were referred for further neuropsychological testing. In the second stage, the neuropsychological test battery Spanish and English Neuropsychological Assessment Scales (SENAS)^35^ and the Informant Questionnaire on Cognitive Decline in the Elderly (IQCODE) were used to determine the need for further neurologic examination on the basis of the following criteria: a score ≥3.40 on the IQCODE and a score below the 10th percentile on ≥1 of the SENAS tests, a score below the 10th percentile on ≥4 SENAS tests, or a score >4.0 on the IQCODE. In the third stage, potential cases of dementia were diagnosed by neurologists and neuropsychologists using the Diagnostic and Statistical Manual of Mental Disorders–IV and National Institute of Neurologic and Communicative Disorders and Stroke‐Alzheimer Disease and Related Disorders Association criteria. Participants were classified as normal, cognitively impaired but not demented, or demented. Participants with dementia were subject to further magnetic resonance imaging and laboratory tests. In this analysis, we combined dementia and CIND cases into 1 outcome.

### Statistical Analyses

Our statistical analysis was focused on assessing the associations of CVD risk and education with cognitive decline and incidence of dementia. That is, our work was focused on interactions of CVD and time and education in models of cognitive function and dementia/CIND. We stratified our analyses by sex because of reported variations in cardiovascular risk between women and men.^17,36^

To estimate the associations of CVD risk with cognitive decline on the 3MSE and SEVLT tests in women and men over the 10‐year period, we used linear mixed models.^37^ We modeled repeated cognitive scores as a function of time, in which time was operationalized as age at cognitive measurement and was grand‐mean‐centered at 70 years. The fitted linear mixed models included main effects for age (as time), CVD risk, and a CVD risk‐by‐age interaction corresponding to the amount of CVD‐related cognitive decline. The models also included random intercepts and slopes. To fulfill the normality assumptions of linear mixed models (Table 3), we examined log‐transformed errors on the 3MSE (101‐3MSE score), with higher errors denoting worse function. To illustrate the CVD risk by age interactions from the fitted linear mixed models, that is, the amount of cognitive decline associated with CVD risk, we estimated the coefficients for age at specific values of CVD risk. We present these estimates at the 25th, 50th, and 75th percentiles of CVD risk, as these are representative of the sample CVD risk distribution. These estimates were back‐transformed and are interpreted as the annual percent change in errors on the 3MSE and annual change in SEVLT scores at the specific values of CVD risk. To evaluate whether the associations of CVD risk with cognitive decline on the 3MSE or SEVLT were modified by education level, we tested for appropriate 3‐way interactions of CVD risk by age by education level.

To estimate the associations of CVD risk with incidence of dementia/CIND in women and men (Table 5), we fitted Cox proportional hazard models. Participants without a dementia/CIND diagnosis by the end of study were right‐censored at the time of their last contact. To evaluate whether the relationship between CVD risk and incidence of dementia/CIND was modified by education level, our Cox models included 2‐way interactions of CVD risk by education level in addition to the main effects for CVD risk and education. To illustrate the magnitude of the CVD risk‐by‐education interactions and their associations with the incidence of dementia/CIND, we present hazard ratios (HRs) and 95% confidence intervals for the associations of education with dementia/CIND at the 25th, 50th, and 75th percentiles of CVD risk. In Figure, we further illustrate the effect modification by education in women by presenting predicted survival functions to dementia/CIND diagnosis at specific values of CVD risk and education. Although CVD risk score included age, we still adjusted for baseline age because of its major role in the development of cognitive impairment and dementia. All statistical analyses were performed using SAS v.9.2.^38^

**Figure 1.**
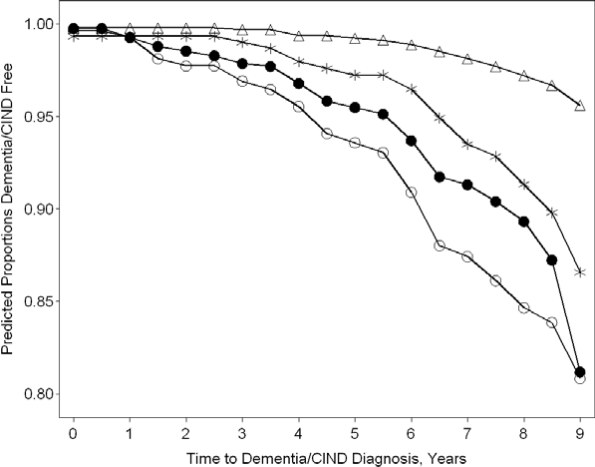
Age‐adjusted predicted proportions of dementia/CIND free in women according to education level and percentile of CVD risk from Cox proportional hazards models. The 4 survival functions correspond to “Education: ≥12 years; CVD risk: 25th percentile” (Triangle), “Education: ≥12 years; CVD risk: 75th percentile” (Star), “Education: <6 years; CVD risk: 25th percentile” (Dot), and “Education: <6 years; CVD risk: 75th percentile” (Circle). CIND indicates cognitive impairment but not dementia; CVD, cardiovascular disease.

## Results

As shown in Table 1, CVD risk score was significantly lower in women (mean, 41.1%; SD, 13.8%) than in men (mean, 45.9%; SD, 14.2%). Women were more likely than men to be “not hypertensive” and showed better cognitive function on the SEVLT but not on the test of global cognitive function (3MSE).Compared with men, women were more likely to be nonsmokers and showed significantly lower SBP but higher mean total cholesterol and HDL (data not shown).

**Table 1.** Sample Characteristics of the Study Population at Baseline by Sex, SALSA 1998–2008

Characteristics	Women, n=663 (59.4%)	Men, n=453 (40.6%)	*P* Value
	No. (%) or Mean (SD)	No. (%) or Mean (SD)	
CVD risk score (0% to 100%)*	41.1 (13.8)	45.9 (14.2)	<0.01
Education (y)			
Low: <6	265 (40.0)	166 (36.6)	0.36
Middle: ≥6 and <12	202 (30.5)	136 (30.0)	
High: ≥12	196 (29.6)	151 (33.3)	
Nativity			
US‐born	305 (46.0)	225 (49.7)	0.25
Mexican‐born	358 (54.0)	228 (50.3)	
Hypertension			
Not hypertensive	301 (45.4)	184 (40.6)	0.03
Hypertensive on meds	217 (32.7)	138 (30.5)	
Hypertensive not on meds	145 (21.9)	131 (28.9)	
3MSE (raw scores)*	86.1 (11.6)	86.9 (9.9)	0.24
SEVLT*	9.5 (2.8)	7.8 (2.8)	<0.01

* SALSA indicates Sacramento Area Latino Study on Aging; SD, standard deviation; CVD, cardiovascular disease; 3MSE, Modified Mini–Mental State Exam; SEVLT, Spanish and English Verbal Learning Test.

Compared with participants included in this analysis, those excluded because of missing data on covariate components of the CVD risk score (n=127) were older, less educated, and more likely to be Mexican‐born, either nonhypertensive or hypertensive but not on medications, and to have worse scores on the 2 cognitive tests. However, participants did not differ on diabetes prevalence and smoking status (data not shown).

Our bivariate analyses (Table 2) showed that for women and men, having higher education and being born in the United States were associated with better 3MSE and SEVLT scores, whereas being older and having higher SBP were associated with worse scores. In women and men, older age, higher SBP, and type 2 diabetes were also associated with greater hazard of dementia/CIND. High education was associated with lower hazard of dementia/CIND in women only.

**Table 2.** Bivariate Associations of Sample Characteristics With Baseline Cognitive Function and Hazard of Dementia/CIND by Sex, SALSA 1998–2008

Characteristics	Women (n=663)				Men (n=453)			
	Baseline Cognitive Function		Dementia/CIND Incidence*		Baseline Cognitive Function		Dementia/CIND Incidence*	
	3MSE (raw scores)*	SEVLT*			3MSE (raw scores)*	SEVLT*		
	Mean (SD) or Pearson *r*	Mean (SD) or Pearson *r*	HR	95% CI	Mean (SD) or Pearson *r*	Mean (SD) or Pearson *r*	HR	95% CI
Age, y*	−0.29*	−0.36*	1.12	1.08 to 1.16	−0.26*	−0.38*	1.10	1.06 to 1.15
SBP, mm Hg*	−0.08*	−0.13*	1.01	1.00 to 1.03	−0.10*	−0.12*	1.02	1.00 to 1.03
Total cholesterol, mg/dl*	0.04	0.05	0.99	0.98 to 1.00	0.06	0.03	1.00	0.99 to 1.01
HDL, mg/dL*	0.09*	0.06	1.00	0.98 to 1.02	−0.05	−0.03	1.02	0.99 to 1.05
Smoking status*								
Nonsmoker	86.61 (10.70)	9.51 (2.84)	1.0	—	87.64 (10.01)	8.11 (2.56)	1.0	—
Former smoker	84.98 (13.60)	9.28 (2.82)	1.03	0.59 to 1.81	87.11 (9.94)	7.82 (2.86)	1.57	0.60 to 4.13
Current smoker	85.60 (11.02)	9.60 (2.83)	1.15	0.45 to 2.90	85.14 (9.50)	7.58 (2.93)	1.96	0.64 to 6.01
BMI, kg/m^2^*	−0.004	0.03	0.97	0.93 to 1.01	0.14*	0.07	0.94	0.87 to 1.02
Diabetes*								
No	86.26 (11.56)	9.51 (2.83)	1.0	—	87.01 (10.17)	7.96 (2.87)	1.0	—
Yes	85.64 (11.65)	9.31 (2.83)	1.96	1.18 to 3.25	86.55 (9.15)	7.53 (2.62)	2.99	1.55 to 5.75
Education, y								
Low: <6	79.02 (13.34)*	8.36 (2.83)*	1.0	—	80.86 (9.93)*	6.79 (2.69)*	1.0	—
Middle: ≥6 and <12	88.55 (7.68)	9.44 (2.48)	0.63	0.35 to 1.12	88.05 (8.19)	7.77 (2.50)	0.65	0.30 to 1.39
High: ≥12	93.13 (5.40)	10.98 (2.46)	0.37	0.19 to 0.72	92.44 (7.32)	9.08 (2.71)	0.43	0.19 to 1.01
Nativity								
US‐born	89.42 (8.81)*	10.10 (92.70)*	1.0	—	89.92 (8.75)*	8.38 (2.85)*	1.0	—
Mexican‐born	83.26 (12.84)	8.91 (2.83)	1.58	0.95 to 2.63	83.88 (10.06)	7.31 (2.66)	1.19	0.62 to 2.30
Hypertension								
Not hypertensive	86.42 (12.00)	9.85 (2.78)*	1.0	—	87.04 (10.13)	8.09 (2.76)	1.0	—
Hypertensive on meds	86.68 (10.66)	9.32 (2.86)	1.11	0.63 to 1.96	86.59 (10.30)	7.67 (2.93)	1.93	0.90 to 4.17
Hypertensive not on meds	84.53 (11.94)	8.84 (2.78)	1.39	0.75 to 2.59	86.95 (9.15)	7.69 (2.72)	1.19	0.49 to 2.88

SALSA indicates Sacramento Area Latino Study on Aging; CIND, cognitive impairment but not dementia; 3MSE, Modified Mini–Mental State Exam; SEVLT, Spanish and English Verbal Learning Test; SD, standard deviation; HR, hazard ratio; CI, confidence interval; SBP, systolic blood pressure; HDL, high‐density lipoprotein; BMI, body mass index.

* *P*<0.05.

* Higher 3MSE and SEVLT scores indicate better cognitive function.

* From a bivariate Cox model.

* Part of the predicted CVD risk score.

In our results of the associations between CVD risk and cognitive decline, there was no effect modification by level of education (ie, the 3‐way interactions of CVD risk by age by education level were not significant and thus were not included in our final models). Our multivariable analyses using linear mixed models showed that higher predicted CVD risk was significantly associated with greater change in errors on the 3MSE in women and men (*P*<0.01 for CVD risk×age interaction denoting CVD‐related cognitive decline). To illustrate the significant interaction and thus the magnitude of cognitive decline, we present the results as annual percent change in 3MSE errors at the specified percentiles of CVD risk (Table 3). In education‐ and nativity‐adjusted models (model 2), the annual error on the 3MSE scores was 0.41% lower for women at the 25th percentile of CVD risk (95% CI, −1.14% to 0.31%), 0.11% higher at the 50th percentile (95% CI, −0.51% to 0.72%), and 0.83% higher at the 75th percentile (95% CI, 0.12% to 1.53%). In education‐adjusted models (model 2), the annual error on the 3MSE was 1.76% lower for men at the 25th percentile of CVD risk (95% CI, −2.78% to −0.73%), 0.96% lower at the 50th percentile (95% CI, −1.81% to −0.10%), and 0.12% higher at the 75th percentile (95% CI, −0.82% to 1.07%).

**Table 3.** Percent Annual Change in Errors on the Modified Mini–Mental State Exam From Linear Mixed Models According to Predicted CVD Risk, by Sex

Predicted CVD Risk	Women (n=663)		Men (n=453)	
	Model 1	Model 2	Model 1	Model 2
	% Change (95% CI)	% Change (95% CI)	% Change (95% CI)	% Change (95% CI)
25th Percentile	−1.08 (−1.86 to −0.30)	−0.41 (−1.14 to 0.31)	−1.83 (−2.93 to −0.74)	−1.76 (−2.78 to −0.73)
50th Percentile	−0.26 (−0.92 to 0.41)	0.11 (−0.51 to 0.72)	−0.87 (−1.79 to 0.05)	−0.96 (−1.81 to −0.10)
75th Percentile	0.89 (0.12 to 1.66)	0.83 (0.12 to 1.53)	0.42 (−0.61 to 1.46)	0.12 (−0.82 to 1.07)

Results are shown as annual percent change in errors on the 3MSE, with higher (positive) percent indicating worse cognitive function (increase in errors). Model 1 is age adjusted; model 2 additionally adjusts for education and nativity. In linear mixed models the CVD risk×age interaction indicates that the rate of cognitive decline differs by CVD risk. In women and men, *P* value for CVD risk×age<0.01. In women, values corresponding to percentiles of CVD risk are 31% (25th percentile), 39% (50th percentile) ,and 50% (75th percentile). In men, the values corresponding to percentiles of CVD risk are 34% (25th percentile), 43% (50th percentile), and 55% (75th percentile). CVD indicates cardiovascular disease; CI, confidence interval.

Our multivariable analyses from linear mixed models showed that higher CVD risk was significantly associated with greater decline in SEVLT scores in women (*P*=0.02 for CVD risk×age interaction denoting CVD‐related cognitive decline) but not in men (*P*=0.1). To illustrate the interaction and the magnitude of cognitive decline, we present the results as annual change in SEVLT scores at the specified percentiles of CVD risk (Table 4). In education‐ and nativity‐adjusted models (model 2), the annual decrease in SEVLT score was 0.09 points for women at the 25th percentile of CVD risk (95% CI, −0.11 to −0.06), 0.10 points at the 50th percentile (95% CI, −0.12 to −0.08), and 0.12 points at the 75th percentile (95% CI, −0.14 to −0.09).

**Table 4.** Annual Change in the Spanish and English Verbal Learning Test Scores From Linear Mixed Models According to Predicted CVD Risk, by Sex

Predicted CVD Risk	Women (n=663)		Men (n=453)	
	Model 1	Model 2	Model 1	Model 2
	Estimate (95% CI)	Estimate (95% CI)	Estimate (95% CI)	Estimate (95% CI)
25th Percentile	−0.08 (−0.10 to −0.05)	−0.09 (−0.11 to −0.06)	−0.03 (−0.07 to 0.01)	−0.03 (−0.06 to 0.01)
50th Percentile	−0.10 (−0.12 to −0.07)	−0.10 (−0.12 to −0.08)	−0.04 (−0.07 to −0.01)	−0.04 (−0.07 to −0.01)
75th Percentile	−0.12 (−0.15 to −0.10)	−0.12 (−0.14 to −0.09)	−0.06 (−0.09 to −0.02)	−0.05 (−0.09 to −0.02)

Results are shown as estimated annual points of decline on the SEVLT associated with percentile predicted CVD risk. Model 1 is age‐adjusted; model 2 additionally adjusts for education and nativity. In linear mixed models, CVD risk×age interaction indicates that the rate of cognitive decline differs by CVD risk. In women, *P* value for CVD risk×age=0.02; in men, *P* value for CVD risk×age=0.1. In women, values corresponding to percentiles of CVD risk are 31% (25th percentile), 39% (50th percentile), and 50% (75th percentile). In men, values corresponding to percentiles of CVD risk are 34% (25th percentile), 43% (50th percentile), and 55% (75th percentile). CVD indicates cardiovascular disease; CI, confidence interval.

In our results of the associations between CVD risk and incidence of dementia/CIND, there was effect modification by education in women. As noted in the footnote of Table 5, the 2‐way interaction of CVD risk by education was significant in women (*P*=0.05) but not in men (*P*=0.9). Results shown in Table 5 are based on fitted age‐adjusted Cox models that include main effects for CVD risk, education, and CVD risk×education interactions. To illustrate this interaction, we present the hazard of dementia/CIND associated with education level at the specified percentiles of CVD risk. Compared with <6 years of education, having ≥12 years of education was associated with a 76% lower hazard of dementia/CIND for women at the 25th percentile of CVD risk (HR, 0.24; 95% CI, 0.08 to 0.71), a 66% lower hazard of dementia/CIND at the 50th percentile (HR, 0.34; 95% CI, 0.15 to 0.79), and a 45% lower hazard of dementia/CIND at the 75th percentile (HR, 0.55; 95% CI, 0.28 to 1.07). For men, the association of CVD risk and dementia/CIND was attenuated by 40% and was nonsignificant after adjusting for education (data not shown). In age‐adjusted Cox models in men, the associations of education with dementia/CIND according to percentiles of CVD risk were all nonsignificant. Compared with <6 years of education, having ≥12 years of education was associated with a 10% lower hazard of dementia/CIND for men at the 25th percentile of CVD risk (HR, 0.90; 95% CI, 0.25 to 3.25), a 25% lower hazard of dementia/CIND at the 50th percentile (HR, 0.75; 95% CI, 0.28 to 2.02), and a 41% lower hazard of dementia/CIND at the 75th percentile (HR, 0.59; 95% CI, 0.22 to 1.61).

**Table 5.** Age‐Adjusted Associations of Education With Dementia/CIND Incidence From Cox Proportional Hazards Models, by Level of Predicted CVD Risk

Predicted CVD Risk	Women (n=663)			Men (n=453)		
	Education (No. of Events/No. at Risk)			Education (No. of Events/No. at Risk)		
	<6 Years (33/265)	6 to 11 Years (18/202)	≥12 Years (12/195)	<6 Years (17/166)	6 to 11 Years (11/136)	≥12 Years (8/151)
	HR (95% CI)	HR (95% CI)	HR (95% CI)	HR (95% CI)	HR (95% CI)	HR (95% CI)
25th Percentile	1	0.68 (0.28 to 1.64)	0.24 (0.08 to 0.71)	1	0.69 (0.18 to 2.65)	0.90 (0.25 to 3.25)
50th Percentile	1	0.70 (0.35 to 1.40)	0.34 (0.15 to 0.79)	1	0.75 (0.27 to 2.11)	0.75 (0.28 to 2.02)
75th Percentile	1	0.74 (0.42 to 1.33)	0.55 (0.28 to 1.07)	1	0.83 (0.38 to 1.82)	0.59 (0.22 to 1.61)

From Cox models, in women, *P* value for CVD risk×continuous education=0.05; in men, *P* value for CVD risk×continuous education=0.9. In women, values corresponding to percentiles of CVD risk are 31% (25th percentile), 39% (50th percentile), and 50% (75th percentile). In men, values corresponding to percentiles of CVD risk are 34% (25th percentile), 43% (50th percentile), and 55% (75th percentile). CIND indicates cognitive impairment but not dementia; CVD, cardiovascular disease; HR, hazard ratio; CI, confidence interval.

To illustrate the CVD risk‐by‐education interaction in women, we present age‐adjusted predicted likelihood of dementia/CIND free (survival functions) based on age‐adjusted Cox proportional hazards models (Figure). The upper survival curves correspond to women with ≥12 years of education at the 25th (CVD risk, 31%) and 75th (CVD risk, 50%) percentiles of CVD risk. The bottom survival curves correspond to women with <6 years of education at the 25th (CVD risk, 34%) and 75th (CVD risk, 55%) percentiles of CVD risk. At either level of education, women with lower CVD risk showed a higher likelihood of dementia/CIND free than women with higher CVD risk. We also found that the decrease in the likelihood of dementia/CIND free (ie, increase in probability of dementia/CIND) at higher CVD risk (75th percentile) was more pronounced in women with ≥12 years of education than in women with <6 years of education (ie, the differential in survival functions associated with increasing CVD risk is bigger in those with ≥12 years of education compared with <6 years). Another way of explaining this interaction is that at lower CVD risk (25th percentile), women with ≥12 years of education had a lower likelihood of dementia/CIND than did women with <6 years of education. However, this protective benefit of having high education relative to low education (ie, the difference between the likelihoods or survival functions) decreased at higher CVD risk (75th percentile).

## Discussion

We have provided evidence that higher predicted CVD risk was associated with greater change in errors on 3MSE, greater decline in SEVLT, and greater hazard of dementia/CIND. These associations were larger and more significant in women than men. In women, education modified the association of CVD risk with dementia/CIND incidence such that the cognitive benefit of having high education decreased as CVD risk increased.

There are several plausible mechanisms by which cardiovascular risk factors may influence cognitive decline and dementia. Insulin resistance and dysregulation associated with diabetes,^39^ increased cerebral perfusion and cortical atrophy associated with high BP,^40^ obesity,^6,41^ and increased production of B‐amyloid or the presence of apolipoprotein 4 allele associated with high cholesterol,^42^ as well as inflammation and oxidative stress associated with smoking,^43–44^ may result in neuronal damage, increased cognitive impairment, and dementia risk.

Our results corroborate evidence from previous studies describing the role of multiple cardiovascular risk factors in shaping cognitive function.^9–13,8,45–46^ There have been only 2 studies that examined dementia as an outcome,^45–46^ neither of which included low‐income minority populations. Having a higher number of nonideal cardiovascular risk factors at midlife, based on a modified version of the Framingham risk score, was associated with greater risk of dementia after 27 years of follow‐up of members of Kaiser Permanente.^46^ Results from a Finnish cohort aged 65 to 79 years also showed an association between multiple cardiovascular risk factors and the odds of dementia at year 20.^45^

Recent findings from the Rancho Bernardo study, an affluent non‐Hispanic white cohort, provided evidence for an association of higher cardiac risk score with cognitive decline among women but not men.^9^ In middle‐aged participants of the Whitehall II, higher CVD risk was associated with greater cognitive decline^8^ more strongly in women than men. Finally, results from the Reasons for Geographic and Racial Differences in Stroke study (REGARDS) also suggested a positive association between the Framingham Stroke Risk Profile (FSRP) and incident cognitive impairment on a test of recall and attention.^10^ Cross‐sectional results from the Framingham Offspring study, a middle‐aged cohort, also showed an association of higher quartile of the FSRP with lower cerebral brain volume^13^ and lower cognitive performance.^11^

In our cohort, the CVD risk–cognitive decline and dementia associations were more significant in women than men. These sex differences have been reported in prior work^8–9^ and, in part, may be because of associated sex differences in vascular physiology. For example, the prevalence of coronary microvascular dysfunction, which may result in microvascular damage in the brain and increased white matter hyperintensities,^47^ may be more common in women than men.^48^ In women, high education was associated with lower hazard of dementia/CIND. However, high education did not provide a protective benefit against dementia/CIND at higher levels of CVD risk. Although we acknowledge that our findings are based on a small number of dementia/CIND events and display uncertainty of the estimates using 95% confidence intervals, the hazard ratio associated with high education increased at higher CVD risk. It is possible that at high levels of CV‐related neurodegeneration, subjects with high education are severely affected because they relied heavily on compensatory processes in delaying dementia/CIND. In men, education was not associated with dementia/CIND at the bivariate level. It may be possible that education has low economic returns in men and is associated with hazardous occupation‐related exposures, the majority agricultural in our cohort, therefore not resulting in cognitive benefit. Approximately 56% of the men with ≥12 years of education had manual occupations including agricultural. A total of 17% of women with ≥12 years of education had manual occupations (this does not include housewives). Although the pattern in men suggests that those with ≥12 years of education have a lower hazard of dementia/CIND at increasing predicted CVD risk, it is difficult to make an inference on the associations between CVD risk and dementia/CIND in men while accounting for education, potentially because of the lack of statistical power.

Most of our ethnic cohort maintained low socioeconomic status across their life course.^22^ As such, our cohort constitutes a high‐risk population with higher risk for cardiovascular risk factors such as obesity^14^ and type 2 diabetes,^15^ compared with non‐Latino whites. Men and women in our cohort had significantly higher CVD risk scores than most other populations studied in the Framingham study and other white populations. For example, in our cohort, men had a median CVD risk score of 43% compared with a 14% median CHD risk for male participants of the Rancho Bernardo study.^9^

Acknowledging the old age of our cohort and the role that age plays in cognitive outcomes, we did a sensitivity analysis to evaluate the extent to which the predicted CVD risk reflected age (data not shown). We calculated a c‐statistic representing the probability that the model discriminated by assigning a higher risk to those who developed dementia/CIND.^49^ The c‐statistic was the same for models in which the CVD risk included or excluded age (0.7 for both sexes), thus corroborating confidence in our predicted CVD risk score. We also performed sensitivity analyses adjusting for hypertension, and the results remained unchanged (data not shown).

Our study has limitations that are worth noting. Because of missing data on the cardiovascular risk score, we excluded 127 participants from this analysis who may have differed from the remaining sample. This may limit the generalizability of our results. Because of the longitudinal nature of our study, attrition from death and dropout of subjects with worse cognitive function and worse cardiovascular risk profiles may have resulted in an attenuation of the associations toward the null. As such, we acknowledge that the effect estimates of cognitive decline are relatively modest, but may be clinically meaningful for subjects with high cardiovascular disease risk. This is of further importance on a population level given the high prevalence of cardiovascular disease and its risk factors. We were not able to examine vascular dementia because of the small number of cases (7%). Finally, although we observed evidence for learning effects in the test of global cognitive function among men with lower CVD risk, the learning effect diminished at higher levels of CVD risk. Learning effects are less of an issue in our cohort given that the cognitive tests were administered only once annually and showed fewer ceiling effects. It has been previously shown that a greater time between tests may decrease learning effects.^32^ Our study has several strengths. It is the first population‐based study to examine the association of CVD risk with cognitive decline and dementia among older Mexican Americans. We have longitudinal data over 10 years with repeated measurements of cognitive function and a thorough multistage dementia diagnosis. While no previous studies have explored the association of CVD risk with dementia/CIND incidence in Latinos, primarily because of lack of such data, the SALSA study provided us with the opportunity to report such associations for the first time.

## Conclusions

Our study is the first to provide evidence for an association of predicted CVD risk with cognitive decline and dementia/CIND incidence in elderly Mexican Americans. Our CVD risk score may provide a useful and convenient tool to identify individuals at greater risk for cognitive decline and dementia development. Targeting subjects at high CVD risk and managing modifiable CVD risk factors is an essential preventive strategy.
